# The predictive value of variables measurable in the ambulance and the development of the Predict Sepsis screening tools: a prospective cohort study

**DOI:** 10.1186/s13049-020-00745-6

**Published:** 2020-06-25

**Authors:** Ulrika Margareta Wallgren, Jan Sjölin, Hans Järnbert-Pettersson, Lisa Kurland

**Affiliations:** 1 Karolinska Institutet, Department of Clinical Science and Education, Söderssjukhuset, Sjukhusbacken 10, 118 83 Stockholm, Sweden; 2 Fisksätra Vårdcentral (Primary Health Care Center), Fisksätra torg 20, 133 41 Saltsjöbaden, Sweden; 3 Department of Medical Sciences, Uppsala University, Akademiska sjukhuset, 751 85 Uppsala, Sweden; 4 Department of Medical Sciences, Örebro University, Campus USÖ, Södra Grev Rosengatan 32, 701 12 Örebro, Sweden

**Keywords:** Sepsis, Screening, Emergency medical services, Prehospital, Emergency care

## Abstract

**Background:**

Despite sepsis being a time critical condition with a high mortality, it is often not identified in a timely fashion. The aim of the current study was to create a screening tool based on bedside measurable variables predictive of sepsis among ambulance patients with infection according to clinical judgment by ambulance personnel.

**Methods:**

Prospective cohort study of 551 adult patients presenting with suspected infection, performed in the ambulance setting of Stockholm during 2017–2018. 18 variables were measured in the ambulance (8 keywords related to medical history, 6 vital signs, 4 point-of-care blood tests, in addition to age, gender, and comorbidity. Logistic regression, area under the curve (AUC) and classification trees were used to study the association with sepsis. The AUC, sensitivity, specificity, predictive values and likelihood ratios were used to evaluate the predictive ability of sepsis screening models.

**Results:**

The six variables with the strongest association with sepsis were: systolic blood pressure ≤ 100 mmHg, temperature > 38.5 °C, GCS < 15, lactate > 4 mmol/L, gastrointestinal symptoms, and a history of acute altered mental status. These were combined into the *Predict Sepsis screening tool 1*, with a sensitivity of 0.90, specificity 0.41, AUC 0.77; 95% confidence interval [CI] 0.73–0.81, PPV 0.52, and NPV 0.86. Combining a history of acute altered mental status with GCS < 15 and excluding lactate in the *Predict Sepsis screening tool 2* did not noticeably affect the AUC. In addition, the AUCs of these models did not differ noticeably when compared to a model including vital signs alone, with novel calculated cut-offs; the *Predict Sepsis screening tool 3*.

**Conclusions:**

Systolic blood pressure ≤ 100 mmHg, temperature > 38.5 °C, GCS < 15, lactate > 4 mmol/L, gastrointestinal symptoms, and a history of acute altered mental status demonstrated the strongest association with sepsis. We present three screening tools to predict sepsis with similar sensitivity. The results indicated no noticeable increase of predictive ability by including symptom-variables and blood tests to a sepsis screening tool in the current study population.

**Trial registration:**

NCT03249597.

## Background

Sepsis is one of the most common medical emergencies and the mortality is high [1–3]. Despite sepsis being a time critical condition, it is often not identified in a timely fashion. Since time-to-treatment is related to patient outcome, early identification is necessary.

More than half of the patients with sepsis are transported to hospital by ambulance [4] and time to treatment is halved when the septic patient is identified by ambulance personnel [5]. Today, identification of the septic patient is based mainly on health care professionals using clinical judgment and this identification rate is inadequately low [6, 7]. Previous studies have demonstrated proof of principle that a screening tool increases the identification of septic patients in both the ambulance and the Emergency Department (ED) settings [6–9].

Existing screening tools are mainly based on vital signs and few are developed for use within the ambulance [8–10]. In addition to a complete lack of prospective studies, these screening tools have inherent problems since vital signs are normal in one third of ambulance patients with severe infections [11] which renders a different approach. We have in a previous study demonstrated that certain keywords related to patients´ medical history recur in ambulance records of septic patients [12]. Eight keywords were particularly common with a prevalence exceeding 20%. However, the specificity of these keywords with respect to sepsis has not yet been studied and information related to patients´ medical history has not previously been included in sepsis screening tools. Additionally, no prior studies have demonstrated the added value of point-of-care (POC) blood tests for sepsis screening within ambulance care with the exception of lactate and glucose [10]. suPAR (soluble urokinase Plasminogen Activator Receptor) and HBP (heparin-binding protein) are two novel biomarkers for sepsis [13, 14] not previously studied in the ambulance.

We hypothesized that the identification of sepsis within ambulance care could be increased by combining keywords related to the patients’ medical history and POC tests in addition to vital signs for sepsis screening. The aim of the current study was to create a screening tool based on bedside measurable variables predictive of sepsis among ambulance patients with infection according to clinical judgment by ambulance personnel. This is, to our knowledge, the first prospective study in the ambulance setting to study the association between symptoms, vital signs and POC tests and the outcome sepsis.

## Methods

### Study design and setting

The current study was a prospective cohort study in the ambulance setting of Stockholm performed between April 3rd, 2017 and August 30th, 2018. The study was performed Stockholm-county-wide, in collaboration with the ambulance provider Samariten Ambulans AB and all seven hospital bound EDs (Södersjukhuset, Karolinska Huddinge, Karolinska Solna, St Göran, Danderyd, Norrtälje, Södertälje) in Stockholm County Council. Samariten Ambulance AB is one of three ambulance providers in Stockholm, and accounts for 75,000 of approximately 183,000 annual ambulance assignments [15, 16]. Ambulances are staffed with two nurse specialists or one nurse specialist and one emergency medical technician [16]. The population of Stockholm county is approximately 2.3 million [17] with approximately 480,000 annual visits to the EDs [18] in the Stockholm City County.

### Selection of study participants

**Fig. 1:**
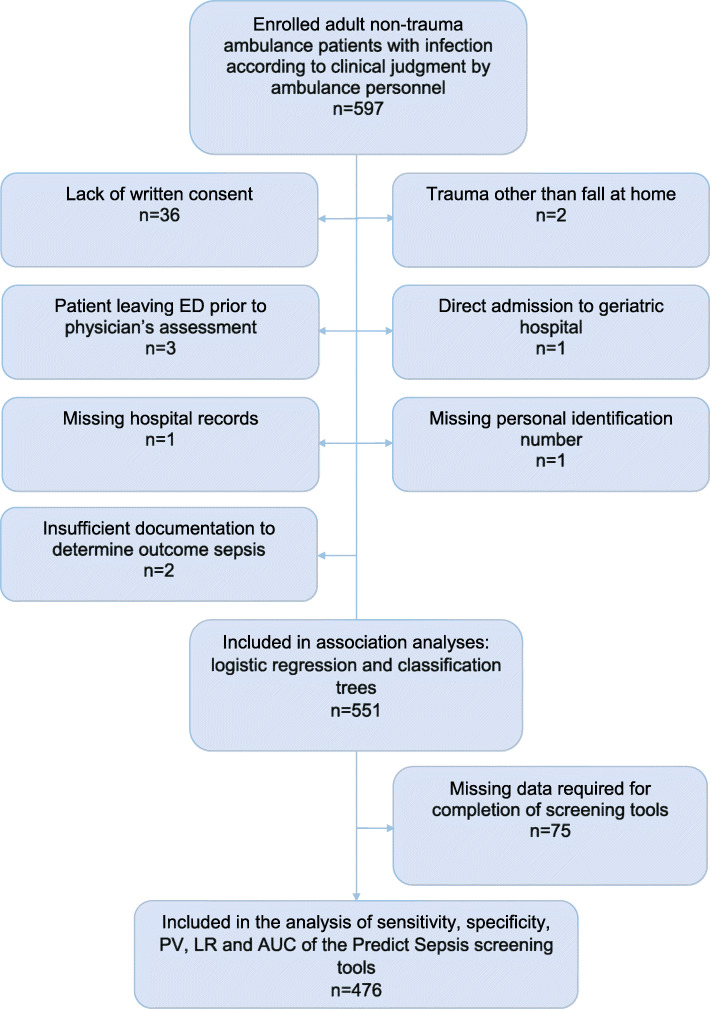
Flow chart of inclusion and exclusion. ED = Emergency Department, PV=Predictive value, LR = Likelihood ratio, AUC = Area under the receiver operating curve

See Fig. 1 for flow chart of inclusion and exclusion.

#### Inclusion criteria

Inclusion criteria were adult (≥18 years) non-trauma ambulance patients considered to suffer from a new onset infection (defined as symptoms that had begun within the last days) according to clinical judgment by the ambulance personnel.

All patients were enrolled by the ambulance personnel and transported to one of the above listed seven hospital bound EDs.

#### Exclusion criteria

Exclusion criteria were: 1) lack of written consent; 2) trauma other than falls at home; 3) patient leaving ED prior to physician assessment; 4) direct admission to geriatric hospital i.e. bypassing an ED; 5) missing hospital records; 6) missing personal identification number; and 7) insufficient documentation to determine outcome sepsis.

### Study protocol

A Case Report Form (CRF) including eight keywords related to medical history and six vital signs was used by the ambulance personnel. Vital signs not recorded in the CRF were extracted from the ambulance records (amPHI® Prehospital ambulance record, Amphi Systems A/S, Aalborg, Denmark, and the digital IT-support for prehospital care in Stockholm; FRAPP® (Framtida IT-plattform för prehospital vård i Stockholms läns landsting).

Data related to ED arrival time, age, gender, pre-existing comorbidity, criteria for suspicion of a new-onset infection, in-hospital vital signs/ laboratory tests/ mortality and discharge International Classification of Diseases (ICD) code were retrieved from the hospital medical records (TakeCare®, v. 18.3.10, CompuGroup Medical, Stockholm, Sweden).

### Predictive variables

A total of 21 variables were measured, as follows:

#### Keywords related to medical history

Eight keywords related to medical history, with a previously demonstrated prevalence exceeding 20% among septic patients in the ambulance based on results from a prior study [12], were registered in the ambulance. These keywords were: “fever or suspected fever”, “pain”, “acute altered mental status”, “weakness of the legs”, “breathing difficulties”, “loss of energy”, “gastrointestinal symptoms” and “risk factors for sepsis” [12], (see Table 1).

**Table 1:** Characteristics of the 551 ambulance patients with suspected infection^a^

**Variable**	**Number**^**b**^**(%**^**b**^**)** ***N*** **= 551**	**Median (IQR)**
**Age (yr)**		78 (71–85)
**Gender**		
-male	331/551 (60.1)	
**Ambulance parameters**		
**Prio**		2 (2–2)
1	100/545 (18.3)	
2	384/545 (70.5)	
3	61/545 (11.2)	
**Prevalence of keywords related to medical history**^c^		
1. Fever or suspected fever	403/551 (73.1)	
2. Pain	256/550 (46.5)	
3. Acute altered mental status	328/551 (59.5)	
4. Weakness of the legs (difficulties to walk/stand/raise/fallen/found on the floor or similar)	419/551 (76.0)	
5. Breathing difficulties	280/549 (51.0)	
6. Loss of energy	490/551 (88.9)	
7. Gastrointestinal symptoms (vomiting/diarrhoea)	188/550 (34.2)	
8. Risk factors for sepsis^d^	229/549 (41.7)	
**Vital signs**		
1. Respiratory rate (min^−1^)		22 (18–28)
2. Oxygen saturation (%)		94 (91–97)
3. Heart rate (min^−1^)		94 (80–108)
4. Systolic blood pressure (mmHg)		135 (120–150)
5. GCS (score)		15 (15–15)
6. Temperature (°C)		38.3 (37.5–39.1)
**POC-tests**		
1. P-Glucose (mmol/L)		7.9 (6.8–9.7)
2. P-Lactate (mmol/L)		1.7 (1.3–2.6)
3. P-suPAR (ng/mL)		4.8 (3.5–6.7)
4. P-HBP (ng/mL)		12.9 (5.9–28.4)
**Comorbidity**		
Charlson comorbidity score		2 (1–4)
**Admitted to in-hospital care**	454/551 (82.4)	
**Outcome**		
1. Sepsis	230/551 (41.7)	
2. No sepsis	321/551 (58.3)	
-Infection no sepsis	277/551 (50.3)	
-No infection	44/551 (8.0)	
**ICD-code upon hospital discharge**		
ICD-code sepsis	54/549 (9.8)	
ICD-code infection	358/549 (65.2)	
**In-hospital mortality**	33/551 (6.0)	

*IQR* Interquartile range, *GCS* Glasgow Coma Scale, *POC* Point Of Care, *suPAR* soluble urokinase Plasminogen Activating Receptor, *HBP* Heparin Binding Protein, *ED* Emergency Department, *qSOFA* quick SOFA (Sequential Organ Failure Assessment score), *ICD* International Statistical Classification of Diseases and Related Health Problems

^a^551 adult ambulance patients with infection according to clinical judgment by ambulance personnel and documentation to determine if the patient had outcome sepsis according to Sepsis-3 or not

^b^Of patients with documented variable

^c^All symptoms were new-onset or increased compared to the patient’s habitual state

^d^such as infection/antibiotic treatment/chemotherapy/ surgical or urological procedure/new blood−/urinary catheters last weeks or alcohol/drug abuse

#### Vital signs

The first measured value in the ambulance of the six vital signs respiratory rate, oxygen saturation, heart rate, systolic blood pressure, Glasgow coma scale (GCS) and temperature were included.

#### POC-tests

Blood was drawn in the ambulance for four POC-tests; P-Glucose, P-Lactate, P-HBP and P-suPAR. For a detailed description of the handling and analyses of these POC-tests see Additional file 1.

#### Demographic variables

Age, gender, and data required for calculation of Charlson comorbidity score [19] were extracted from hospital records. Charlson comorbidity score is a validated method used to classify comorbid conditions which influence the risk of mortality and is developed for use in longitudinal studies [19].

### Outcomes

The possible outcomes were sepsis or no sepsis, within the first 36 h after ED arrival.

#### Sepsis

Sepsis was defined as sepsis within 36 h from ED arrival, in accordance with the Sepsis-3 criteria [20]; i.e. infection (as defined in Additional file 2) in combination with an increased Sequential Organ Failure Assessment (SOFA) score of 2 points or more, as compared with the patient’s preexisting status and based on review of the medical record. Septic shock was defined as vasopressor requirement and serum lactate level greater than 2 mmol/L [20]. The preexisting score was set to zero for patients with no previous recordings of variables needed for calculating the SOFA score [20]. Oxygen saturation level and level of oxygen supplied were converted to the partial pressure of oxygen (PaO2)/fraction of inspired oxygen (FiO2) in accordance with Swedish Intensive care registry [21, 22].

#### No sepsis

Patients that did not fulfill sepsis criteria, as described above, were classified as “no sepsis”.

### Calculation of sample size

The current study was the first part of the larger Predict Sepsis study (NCT03249597). The sample size for the current study was originally based on 18 variables to be used in the logistic regression analysis which implied that 180 patients with sepsis were needed i.e. ten events for each predictor variable [23]. 20% additional patients were included to compensate for missing data. Thus, the recruitment goal was set to include 216 patients with outcome sepsis (NCT03249597).

The prevalence of sepsis among ambulance patients was not previously known. Therefore, the first enrolled 315 patients were used to estimate the prevalence of sepsis in the study population and for the calculation of the final sample size.

### Data analysis

Statistical analyses were performed using SPSS (Statistical Package for the Social Sciences) statistical software v. 23–25.0 (SPSS Inc., Chicago, IL, USA), and Clinical Research Calculators; Calculator 1, Vassarstats.net [24].

#### Characteristics

Normality distribution was assessed with the Kolmogorov–Smirnov and the Shapiro–Wilk tests and visually in histograms. Median and interquartile range (IQR) were used to describe age, vital signs and POC-test levels, since these variables were not normally distributed.

#### Classification of variables in the regression analysis

##### Keywords related to medical history

Keywords were classified as present (yes)/ not present (no) in the regression analysis. Patients not able to answer yes or no were included in the yes-category for the association analyses since they were few (11–30 patients per keyword) and the prevalence of sepsis was similar.

##### Vital sign and POC-test categories

Categories for numerical variables (vital signs and POC-tests) were calculated using a stepwise approach, including the following steps:

1. 8–10 categories were created for each numerical variable, including previously defined categories according to NEWS [25], SIRS [26], Robson [10] when possible, and aiming for equal ranges for each created category.
2. The 8–10 categories from step 1 were merged into 3–4 categories for each variable, based on odds ratios, with the aim to not overlap the 95% confidence intervals and that the prevalence of sepsis was similar in the merged categories.
3. The 3–4 categories from step 2 were merged into the final 2–3 categories using the same criteria as above described in step 2.

Comparisons of receiver operating characteristic (ROC) curves and AUC values for the continuous variable and their three categorized versions (8–10 categories, 3–4 categories and 2–3 categories for each numerical variable) were performed for each step in order to assess that the categorization had not caused an inacceptable loss of information.

Underlying data describing the prevalence of sepsis within categories is described in Additional file 3.

#### Determination of predictors of sepsis among patients with infection in the ambulance according to clinical judgment by ambulance personnel

##### Logistic regression

Identification of predictor variables for sepsis was performed as follows: first an unadjusted univariable (crude) analysis was performed for each of the 21 variables. AUC values were calculated for all variables that showed a significant association (*p* < 0.05) with sepsis. Second, a multivariable adjusted logistic regression was performed including variables which were significantly associated with outcome sepsis in the univariable analysis. Odds ratios (ORs) were reported with corresponding 95% confidence intervals.

##### Classification trees

Classification trees were used as a complement to logistic regression to identify variables associated with sepsis and to stratify groups of patients according to risk of sepsis. An advantage of this method as compared to logistic regression is that interactions between variables can be discovered and visualized. The Chi-squared automatic interaction detection (CHAID) algorithm was used to build the trees [27] and starts with all data in one group. Each possible split for each variable is considered in order to find the split that leads to the strongest association with the outcome: i.e. sepsis (yes/no). The analysis was based on the 21 variables described above. The resulting groups were split until one of the following stop criteria was reached: tree depth was limited to five levels, a group with less than 25 patients was formed or a split with a Bonferroni adjustment of less than 0.05 was executed.

#### Models used to predict sepsis

Models for sepsis screening were created based on significant association with sepsis in univariable and multivariable regression analyses, in addition to significant association in univariable analysis in combination with significant *p*-values (< 0.05) for the AUC of the variable.

There was a trade-off between the number of variables included in the model and the contribution to prediction of the outcome sepsis. The objective to include a small number of variables rather than a larger is based on the assumption that the screening tool is a clinical bedside tool vs an electronically embedded tool. As a final step, models combining the keyword acute altered mental status and GCS < 15 and, additionally, models excluding lactate were tested. This was done to evaluate, by ROC curves, how reduction of variables and avoidance of an invasive step (lactate measurement) affected the predictive ability with respect to sepsis identification.

The predictive models were evaluated based on scores for individual variables instead of the estimated regression scores, as follows. First, each individual variable in the model was scored based on the strength of the association with sepsis in regression analyses and classification trees. Secondly, cut-offs for total scores were evaluated with respect to sensitivity and specificity for sepsis by applying ROC curves. A comparison of the predictive ability for each model, given a specific cut-off for total score, was performed by calculating AUC (according to SPSS), sensitivity, specificity, positive predictive value (PPV), negative predictive value (NPV) and likelihood ratios (LRs) (according to Vassarstat.net [24]).

### Ethical approval and compliance with international standards of study procedures

The study received approval from the Stockholm Regional Ethical Review Board (reference number 2016/2001–31/2 and 2018/2202). Written consent was obtained from all participants.

This study complied with the Declaration of Helsinki [28] and the manuscript was drafted according to the Standards for the Reporting of Diagnostic accuracy studies (STARD) criteria [29].

## Results

### Patient characteristics

See flow chart for inclusion and exclusion in Fig. 1.

553 patients with suspected infection in the ambulance were included. Of these, 454 (82.4%) were admitted to in-hospital care. A total of 551 patients had sufficient documentation to determine whether the patient had outcome sepsis or not within 36 h from ED arrival and were accordingly included in the regression and classification tree analyses. Patient characteristics are presented in Table 1.

Of the 551 included patients 331 (60.1%) were men, the median age was 78 years (IQR 71–85) and 230 (41.7%) had sepsis, see Table 1. Of the 230 patients with sepsis, 23 patients (10%) died during in-hospital care. Forty-four of 228 patients (19.3%) with sepsis in accordance with the Sepsis-3 criteria [20], and a documented ICD-code upon hospital discharge, had an ICD-code consistent with sepsis.

### Predictors of sepsis

#### Logistic regression analysis

Individual ROC curves for the 17 variables that showed a significant association with sepsis in the univariable analysis are presented in Additional file 4.

##### Keywords related to medical history

The keywords with the strongest association with sepsis were acute altered mental status and gastrointestinal symptoms, see Table 2.

**Table 2:** Association between 21 variables and sepsis^a^ among 551^b^ ambulance patients with suspected infection

**Variable**	**Category**	**Crude**		**Univariable, unadjusted**			**Multivariable, adjusted**		
		***n* = 551**		***P*-value**	***n* = 551**		**Adjusted for all factors that were significant in the univariable analysis,*n* = 484**		
		**n**^**c**^	**% sepsis**		**OR**	**95% CI**	***P*-value**	**OR**	**95% CI**
Keywords related to medical history									
Fever or suspected fever	yes^d^	417	45.1	**0.005**	1.8	1.2–2.7	0.98	1.0	0.6–1.7
	no	134	31.3	Ref	Ref	Ref	–	Ref	–
Pain	yes^d^	277	43.0	0.53	1.1	0.8–1.6	–	–	–
	no	273	40.3	Ref	Ref	Ref	–	Ref	–
Acute altered mental status	yes^d^	344	49.4	**< 0.001**	2.4	1.7–3.5	**0.03**	1.8	1.1–2.9
	no	207	29.0	Ref	Ref	Ref	–	Ref	–
Weakness of the legs	yes^d^	442	44.6	**0.007**	1.9	1.2–2.9	0.61	1.2	0.6–2.1
	no	109	30.3	Ref	Ref	Ref	–	Ref	–
Breathing difficulties	yes^d^	299	48.2	**0.001**	1.8	1.3–2.6	0.24	1.3	0.8–2.2
	no	250	34.0	Ref	Ref	Ref	–	Ref	–
Loss of energy	yes^d^	501	43.1	**0.04**	2.0	1.0–3.7	0.79	1.1	0.5–2.6
	no	50	28.0	Ref	Ref	Ref	–	Ref	–
Gastrointestinal symptoms	yes^d^	214	52.8	**< 0.001**	2.1	1.5–2.3.0	**0.006**	1.9	1.2–2.9
	no	336	34.8	Ref	Ref	Ref	–	Ref	–
Risk factors for sepsis	yes^d^	256	47.7	**0.008**	1.6	1.1–2.2	0.46	1.2	0.8–1.9
	no	293	36.5	Ref	Ref	Ref	–	Ref	–
Vital signs									
Respiratory rate > 24 breaths/min	yes	189	56.1	**< 0.001**	2.4	1.7–3.5	0.53	1.2	0.7–2.0
	no	361	34.3	Ref	Ref	Ref	–	Ref	–
Oxygen saturation < 94%	yes	232	55.6	**< 0.001**	2.8	1.9–3.9	0.08	1.6	1.0–2.5
	no	319	31.2	Ref	Ref	Ref	–	Ref	–
Heart rate > 110 beats/min	yes	106	58.1	**< 0.001**	2.3	1.5–3.5	0.09	1.6	0.9–2.8
	no	446	37.8	Ref	Ref	Ref	–	Ref	–
Systolic blood pressure ≤ 100 mmHg	yes	54	68.5	**< 0.001**	3.4	1.9–6.3	**0.001**	3.6	1.7–7.6
	no	496	38.7	Ref	Ref	Ref	–	Ref	–
Level of consciousness, GCS <15	yes	115	67.0	**< 0.001**	3.8	2.4–5.8	**< 0.001**	3.5	2.0–6.2
	no	416	35.0	Ref	Ref	Ref	–	Ref	–
Temperature, °C							**< 0.001**		
≤ 38.0	yes	235	27.0	Ref	Ref	Ref	–	Ref	–
38.1–38.5	yes	91	41.8	**0.01**	1.9	1.2–3.2	**0.02**	2.2	1.1–4.2
> 38.5	yes	223	56.1	**< 0.001**	3.4	2.3–5.1	**< 0.001**	3.3	2.0–5.6
POC-tests									
P-Glucose > 6.5 mmol/L	yes	410	42.7	0.10	1.5	0.9–2.3	–	–	–
	no	105	33.7	Ref	Ref	Ref	–	Ref	–
P-Lactate, mmol/L							0.08		–
≤ 2.0	yes	340	36.1	Ref	Ref	Ref	–	Ref	
2.1–4.0	yes	160	46.3	**0.03**	1.5	1.0–2.2	0.35	1.3	0.8–2.1
> 4.0	yes	38	76.3	**< 0.001**	5.7	2.6–12.4	**0.03**	2.8	1.1–7.3
P-suPAR, ng/mL							0.31		
< 4.0	yes	184	30.4	Ref	Ref	Ref	–	Ref	–
4.0–7.99	yes	263	41.4	**0.02**	1.6	1.1–2.4	0.41	1.2	0.8–2.0
≥ 8.0	yes	93	64.5	**< 0.001**	4.2	2.4–7.1	0.13	1.7	0.9–3.4
P-HBP ≥15.0 ng/mL	yes	235	52.8	**< 0.001**	2.3	1.6–3.2	0.37	1.2	0.8–2.0
	no	290	33.1	Ref	Ref	Ref	–	Ref	–
Demographic variables									
Age ≥ 65 years	yes	474	41.6	0.83	0.9	0.6–1.5	–	–	–
	no	77	42.9	Ref	Ref	Ref	–	Ref	–
Gender, male	yes	331	43.8	0.23	1.2	0.9–1.8	–	–	–
	no	220	38.6	Ref	Ref	Ref	–	Ref	–
Charlson comorbidity score ≥ 5 points	yes	86	55.8	**0.004**	2.0	1.2–3.1	0.13	1.6	0.9–3.1
	no	465	39.1	Ref	Ref	Ref	–	Ref	–

*OR* Odds Ratio, *CI* Confidence Interval, *Ref* Reference, *GCS* Glasgow Coma Scale, *suPAR* soluble urokinase Plasminogen Activating Receptor, *HBP* Heparin Binding Protein

^a^Sepsis is defined in accordance with Sepsis-3 as a) infection + ≥2 SOFA criteria, or b) infection + vasopressor need and lactate > 2 (septic shock)

^b^Of total 553 patients with infection according to clinical judgment by ambulance personnel, 551 patients had the required documentation to determine whether the patient had sepsis or not. These 551 patients were included in the regression analysis

^c^of patients with documentation of the variable

^d^Patients that were not able to answer yes or no were included in the yes-category in the logistic regression and classification tree analyses (11–30 patients/keyword), based on similarity in prevalence of sepsis and overlapping CIs in these groups

Significant *P*-values are bolded

##### Vital signs

The calculated vital sign and POC-test level categories used in the regression analyses are presented in Table 2. The vital signs with the strongest association with sepsis were systolic blood pressure ≤ 100 mmHg, GCS < 15, and temperature > 38.5 °C, see Table 2. Heart rate demonstrated the weakest association with sepsis.

##### POC-tests

All POC-tests except for P-Glucose had a significant association with sepsis in the univariable logistic regression. The only POC-test that remained significantly associated with sepsis in the multivariable analysis was P-Lactate > 4 mmol/L, see Table 2.

##### Demographic variables

A Charlson comorbidity score of ≥5 points was significantly associated with outcome sepsis in univariable analysis. This association did not remain significant after adjusting for all other variables, see Table 2.

#### Classification trees

**Fig. 2:**
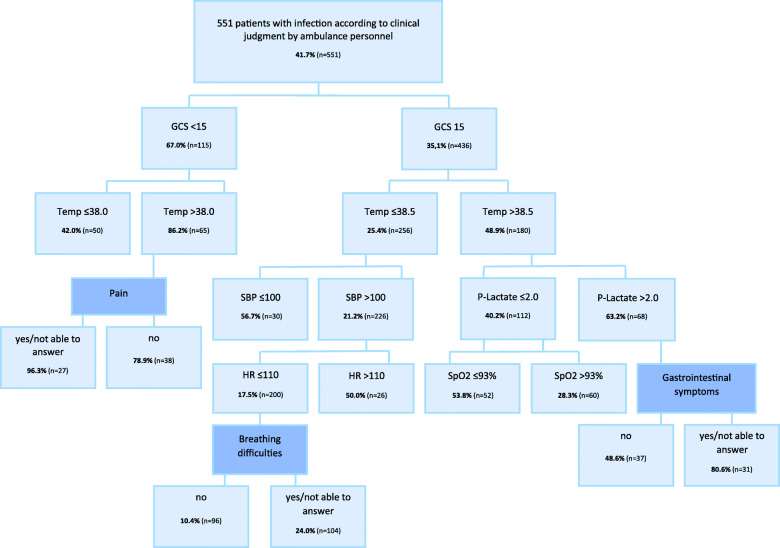
Classification tree^α^ presenting the variables which, at each step, had the strongest association with sepsis*. GCS = Glasgow Coma Scale, Temp = Temperature, SBP=Systolic Blood Pressure, HR = Heart Rate, SpO2 = Saturation of peripheral Oxygen, CHAID = Chi-square Automatic Interaction Detector. ^α^The CHAID algorithm was used to build the tree. *among 551 adult patients with infection according to clinical judgment by ambulance personnel and documentation of to determine whether the patient had sepsis or not. The prevalence of sepsis is bolded and calculated based on the total number of patients in each node (n). Darker blue filling of the box indicates a keyword reflecting medical history. Interpretation; example: “Of the 115 patients with a decreased level of consciousness (GCS<15), 67% had sepsis. If the patients also had fever (Temp>38.0°C), the prevalence of sepsis increased to 86% of the 65 in this group”. All Bonferroni-adjusted values were < 0.05 for all nodes

The vital signs GCS and temperature were most strongly associated with sepsis according to classification tree analyses, as shown in Fig. 2.

### Models and the Predict Sepsis screening tools

**Fig. 3:**
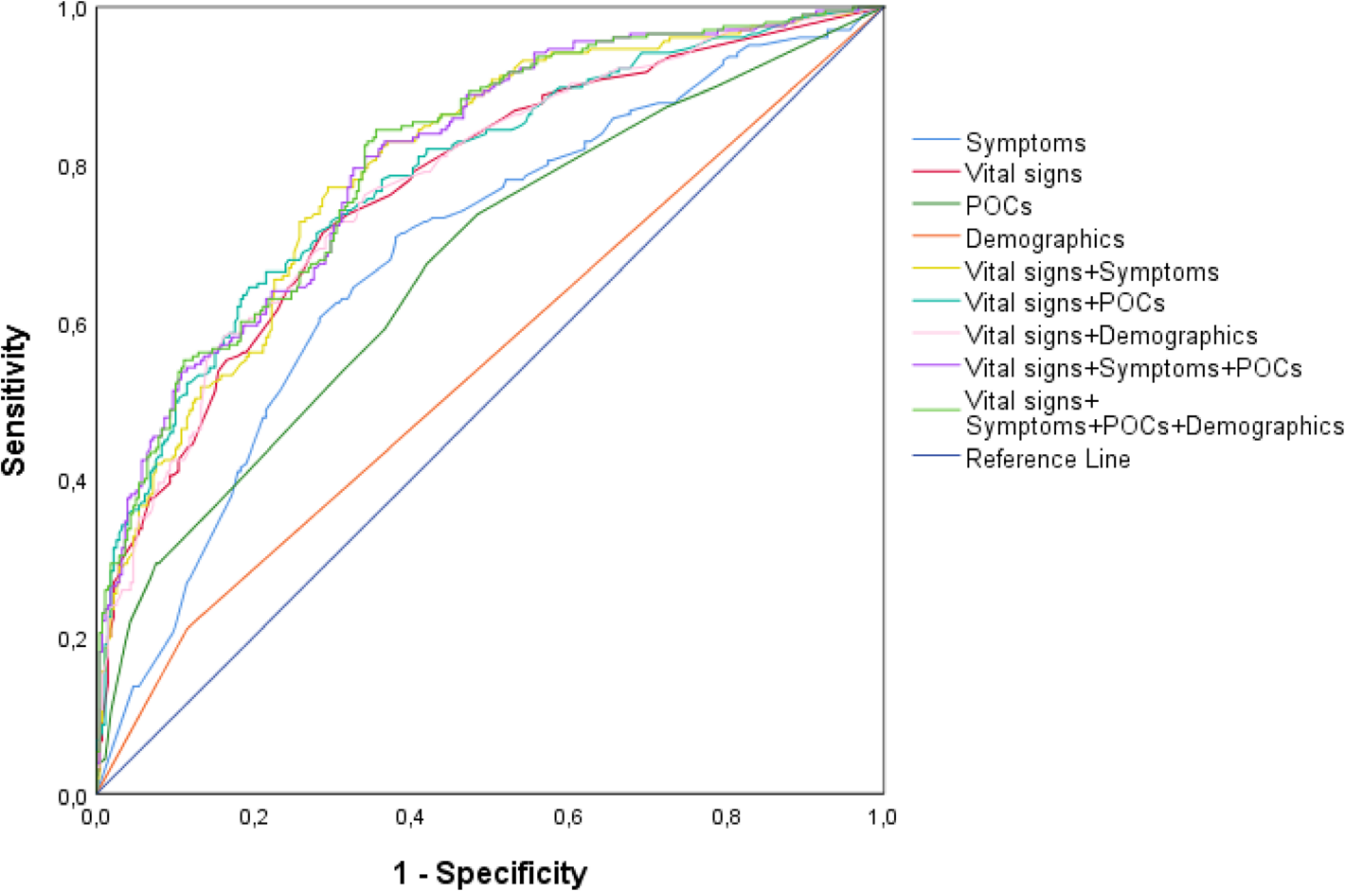
ROC curves for models based on variable groups and combinations of these. ROC = Receiver Operating Characteristic, POCs = point-of-care blood tests. Only variables significantly associated with sepsis in the univariable analysis are included in the models

ROC curves for models based on variable groups (symptoms, vital signs and POC-tests) and combinations thereof are illustrated in Fig. 3. Vital signs were, as a variable group, the strongest predictors of sepsis (Fig. 3).

**Fig. 4:**
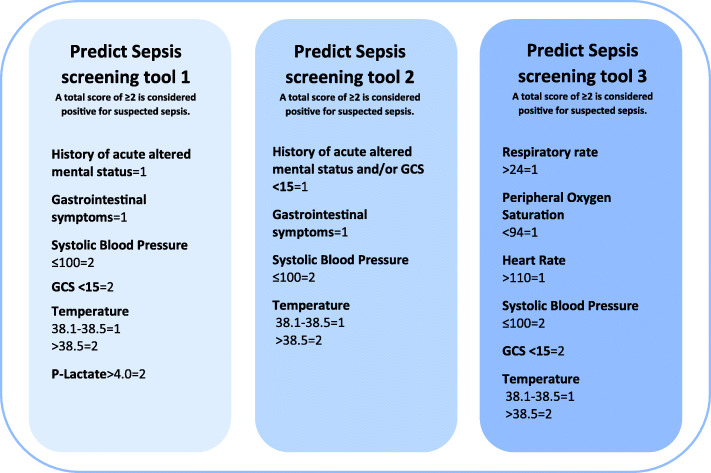
The Predict Sepsis screening tools. The Predict Sepsis screening tools are developed for adult, non-trauma patients with suspected infection according to clinical judgment by ambulance personnel

A description of the variables included in the models and the method used to develop each model is presented in Additional file 5. Scores for individual variables in the tools, and total scores for the models, considered positive for suspected sepsis, were chosen based on comparisons of AUC values, see Table 3 and Fig. 4.

**Table 3:** Sensitivity, specificity, predictive values, likelihood ratios and AUCs of the final models*

Model	**Predict Sepsis screening tool 1**	Model 5	Model 6	Model 7	**Predict Sepsis screening tool 2**	**Predict Sepsis screening tool 3**
Included variables and their scores	*Acute altered mental status = 1* *Gastrointestinal symptoms = 1* SBP ≤ 100 = 2 GCS < 15 = 2 Temp 38.1–38.5 = 1 >38.5 = 2 Lactate> 4 = 2	*Acute altered mental status and/or GCS < 15 = 2* *Gastrointestinal symptoms* = 1 SBP ≤ 100 = 2 Temp 38.1–38.5 = 1 > 38.5 = 2 Lactate> 4 = 2	*Acute altered mental status and/or GCS < 15 = 2* *Gastrointestinal symptoms* = 1 SBP ≤ 100 = 2 Temp 38.1–38.5 = 1 > 38.5 = 2	*Acute altered mental status and/or GCS < 15 = 1* *Gastrointestinal symptoms* = 1 SBP ≤ 100 = 2 Temp 38.1–38.5 = 1 > 38.5 = 2 Lactate> 4 = 2	*Acute altered mental status and/or GCS < 15 = 1* *Gastrointestinal symptoms* = 1 SBP ≤ 100 = 2 Temp 38.1–38.5 = 1 > 38.5 = 2	RR > 24 = 1 SpO2 < 94 = 1 HR > 110 = 1 SBP ≤ 100 = 2 GCS <15 = 2 Temp 38.1–38.5 = 1 > 38.5 = 2
Total score considered positive for suspected sepsis	≥2	≥2	≥2	≥2	≥2	≥2
Sensitivity (95%CI)	0.90 (0.85–0.94)	0.92 (0.88–0.96)	0.92 (0.87–0.95)	0.87 (0.82–0.92)	0.86 (0.80–0.90)	0.91 (0.86–0.94)
Specificity (95%CI)	0.41 (0.35–0.47)	0.27 (0.22–0.33)	0.27 (0.22–0.33)	0.48 (0.42–0.54)	0.48 (0.42–0.54)	0.38 (0.33–0.44)
PPV (95%CI)	0.52 (0.47–0.58)	0.48 (0.43.0.53)	0.48 (0.43–0.53)	0.55 (0.49–0.60)	0.54 (0.49–0.60)	0.51 (0.46–0.57)
NPV (95%CI)	0.86 (0.78–0.91)	0.84 (0.74–0.90)	0.83 (0.73–0.89)	0.84 (0.77–0.89)	0.83 (0.76–0.88)	0.85 (0.78–0.91)
Pos LR (95%CI)	1.54 (1.38–1.71)	1.27 (1.17–1.38)	1.27 (1.17–1.38)	1.67 (1.48–1.89)	1.65 (1.46–1.88)	1.47 (1.33–1.63)
Neg LR (95%CI)	0.23 (0.15–0.36)	0.27 (0.17–0.46)	0.29 (0.18–0.48)	0.26 (0.18–0.38)	0.29 (0.21–0.42)	0.24 (0.15–0.37)
AUC for sum of scores for the model (95%CI)	0.77 (0.73–0.81)	0.75 (0.70–0.79)	0.74 (0.69–0.78)	0.75 (0.70–0.79)	0.74 (0.69–0.78)	0.76 (0.72–0.81)

*PPV* positive predictive value, *NPV* positive predictive value, *LR* likelihood ratio, *AUC* area under the curve, *CI* confidence interval, *SBP* Systolic blood pressure, *GCS* Glasgow coma scale, *Temp* Temperature

*Sensitivity, specificity, PPV, NPV, LRs and 95% confidence intervals for those were calculated by using www.vassarstats.net, Clinical research calculators, calculator 1

*Curved text indicates a keyword related to medical history*

Bold text indicates models chosen as the Predict Sepsis screening tools

The variables demonstrating the strongest association with sepsis according to logistic regression and classification trees were acute altered mental status, gastrointestinal symptoms, systolic blood pressure ≤ 100 mmHg, GCS < 15, temperature > 38.5 °C and P-Lactate > 4 mmol/L. These variables were combined into the “*Predict Sepsis screening tool 1*”, see Table 3 and Fig. 4.

The AUC was not reduced noticeably when a combined variable for a decreased level of consciousness (a history of acute altered mental status and/or GCS < 15) was introduced and P-Lactate was excluded in the *Predict Sepsis screening tool* 2, based on 4 variables (see Table 3 and Fig. 4).

The *Predict Sepsis screening tool 3* was based on the 6 vital signs significantly associated with sepsis in univariable analysis, applying the novel calculated categories for each vital sign (see Table 3 and Fig. 4).

The Predict Sepsis screening tool 1, 2 and 3 identified the same septic patients to a large extent; 163 patients were identified by all three tools, eight patients were identified by tool 1 and 2 but not by tool 3, eight patients were identified by tool 1 and 3 but not by tool 2, one patient was identified solely by tool 1 and nine septic patients were not identified by either tool.

For AUCs of the *Predict Sepsis screening tools* before and after introduction of scores, see Additional file 5 and Table 3. The sensitivity, specificity, predictive values and likelihood ratios for the Predict Sepsis screening tools are presented in Table 3.

## Discussion

This is the first prospective study in the ambulance setting to study the association between variables measurable in the ambulance and the outcome sepsis. It is also the first study to include symptom-variables in a sepsis screening tool. Systolic blood pressure ≤ 100 mmHg, temperature > 38.5 °C, GCS < 15, P-Lactate > 4 mmol/L, gastrointestinal symptoms, and a history of acute altered mental status demonstrated the strongest association with sepsis. I.e. two symptoms and one POC-test were significantly associated with outcome sepsis in the multivariable analysis. However, interestingly, vital signs were, as a variable group, the strongest predictors of sepsis.

The *Predict Sepsis screening tool 1 and 2* both include symptom-variables. The second tool is based on only four variables, which makes it feasible to use as a hands-on screening tool without the need of incorporation in electronic systems. However, the predictive ability of the *Predict Sepsis screening tool 1 and 2* was similar to that of the *Predict Sepsis screening tool 3,* which was built on vital signs alone, but with new calculated cut-offs for each included vital sign. This implies that addition of symptom-variables and POC-tests did not noticeably increase the predictive ability of a sepsis screening tool in the current study population.

It is, in our opinion, the clinical setting where the tool is going to be implemented that is the determining factor for which tool to recommend, since the predictive ability of these three tools was similar.

### Predictors of sepsis

Keywords reflecting gastrointestinal symptoms and acute altered mental status demonstrated a stronger association with sepsis than “classic” symptoms of sepsis such as a history of fever. This finding is novel and indicates that these symptoms require more attention.

Systolic blood pressure, GCS and temperature were the vital signs that demonstrated the strongest association with sepsis. However, one third of the septic patient presented with a normal systolic blood pressure, two thirds had a normal GCS and one third lacked fever. This is consistent with a previous study by Suffoletto et al [11], demonstrating that more than one third of the patients with severe infections present with normal vital signs to the ambulance.

P-Lactate was the POC-test that demonstrated the strongest association with sepsis and was included in some of the models. However, excluding P-Lactate in the *Predict Sepsis screening tool 2* did not noticeably decrease the predictive ability. This raises the question if the benefit of a slightly higher AUC in a screening tool is worth the disadvantages of a clinically invasive step, i.e. a blood test. A previous study by Singer et al. demonstrated a moderate to good specificity but a low sensitivity for POC lactate in adult ED patients with suspected sepsis [30], and Moran et al. stated that the lactate-added value is dependent on the underlying predictive model [31].

Age has been shown to be a predictor of sepsis among ambulance patients [9]. This was however not confirmed in the current study, which could be explained by the median age being similar in the two outcome groups i.e. sepsis and no sepsis.

### Models and comparison of screening tools

All the calculated models demonstrated good AUC values. The inclusion of fewer variables in the models did not noticeably affect the AUC. This is valuable information since the ambulance setting constitutes an environment where every minute counts and using fewer variables should save time, i.e. unless the screening tool can be included in an electronic decision support system. If a screening tool is incorporated in an electronic system, the number of included variables is of less importance. Hence, which tool is the optimal tool will depend on how the tool is planned to be implemented within clinical practice, i.e. as a hands-on screening tool or as an electronic decision support tool.

It is a challenge to develop a tool combining a high sensitivity with a high specificity. The low specificity of both the Predict Sepsis screening tools and preexisting screening tools [8, 10] is troublesome since it may cause false sepsis alerts. However, we considered a high sensitivity to be more important since the major clinical problem is not identifying the septic patient in a timely fashion [5, 6, 32]. Some of the difficulties in combining a high sensitivity with a high specificity could be explained by the heterogenous presentations of sepsis. Sepsis is likely not to be one but rather several conditions influenced by both microbial and host factors, which may contribute to the heterogenicity with respect to presentation. This line of reasoning is supported by Seymour et al., describing several phenotypes of sepsis [33].

### Clinical implementation of the Predict Sepsis screening tools

The objective of the application of a screening tool in the ambulance is to increase the identification of septic patients and enable timely treatment. Previous studies have shown that time to treatment is halved when the septic patient is identified by ambulance personnel [5] underscoring the importance of identification of the septic patient in the ambulance.

The Predict Sepsis screening tool 1 includes lactate measurement, which is not implemented in all ambulance settings. P-Lactate > 4 mmol/L was one of the variables that remained significantly associated with sepsis in the multivariable logistic regression analysis. However, the predictive ability of the models including lactate was not superior to that of the models excluding this invasive step. Hence, we do not consider lactate measurement justifiable as it may cause a delay of the sepsis screening and the transport of the patient in addition to discomfort from the patient’s perspective.

Predict Sepsis screening tool 2 is based on four variables of which two are vital signs and two symptom-based variables. This makes the Predict Sepsis screening tool 2 feasible to use as a hands-on screening tool in an ambulance setting without a computer-based alert system. However, implementation of the Predict Sepsis screening tool 2 requires the ambulance personnel to ask all patients with clinically suspected infection whether they have gastrointestinal symptoms or not. Since this request is not part of standard procedure, it could be considered a disadvantage.

Predict Sepsis screening tool 3 is based on six vital signs alone. The advantage of this tool is that vital signs are routinely measured within ambulance care. It is however noteworthy that the cut-offs for each vital sign were calculated in the current study and not those typically applied today as clinical routine. The application of Predict Sepsis screening tool 3 is particularly feasible in settings with computer-based alert systems, although the tool may also be used manually.

### Strengths and limitations of the current study

The strengths of the current study were the prospective design, the novel concept to include keywords related to medical history and POCs in the analyses, in addition to vital signs, and to apply calculated cut-offs for vital signs and POC-tests rather than using previously published cut-off levels. Patients with clinically suspected infection are common in the ambulance and it is of importance to identify those at risk of developing a severe infection such as sepsis. In addition, patients with a decreased level of consciousness were included in the current study, reducing selection bias.

There are several limitations to the current study. The categorization of vital signs and POC-tests could be questioned. However, the categorized variables followed the ROC curves for the continuous variables to a large extent, supporting well-chosen cut-off levels.

The definition of infection could be criticized. The Sepsis-1 and -2 consensus documents [26, 34] defined infection as “a pathological process caused by invasion of normally sterile tissue/fluid or body cavity by pathogenic or potentially pathogenic micro-organisms”. Neither does Sepsis-3 include a detailed definition of infection [20], nor are there other consensus criteria for infection. The definition of infection used in the current study is based on clinical experience and symptoms frequently reported by patients suffering from infection and has been used in prior publications [6, 7, 12]. The ability of ambulance personnel to identify patients with infection was high in the current study; 92% of the patients with suspected infection according to clinical judgment by ambulance personnel fulfilled the predefined criteria for infection. We consider it an advantage to have used stringent criteria for the definition of infection despite potential shortcomings of this specific definition.

Furthermore, there is an inherent risk that the predictive ability of a screening tool is higher in the population in which it is was developed than in other populations. Hence, the Predict Sepsis screening tools need to be externally validated.

Finally, the results are limited to the specific population of ambulance patients with suspected infection, rendering generalization of the results to the population of all patients presenting to the ambulance as not correct. It would be of interest to study the potential benefit of adding symptom-variables and POC-tests in other study populations; i.e. not only among those with obvious signs of ongoing infection but rather among patients with non-specific presentations, as these patients are at a higher risk of not being identified as being septic.

The developed screening tools require external validation before clinical implementation and are applicable to adult, non-trauma ambulance patients with suspected infection according to clinical judgment by ambulance personnel.

## Conclusions

Systolic blood pressure ≤ 100 mmHg, temperature > 38.5 °C, GCS < 15, lactate > 4 mmol/L, gastrointestinal symptoms, and a history of acute altered mental status demonstrated the strongest association with sepsis. We present three screening tools to predict sepsis with similar sensitivity. The results indicated no noticeable increase of predictive ability by including symptom-variables and blood tests to a sepsis screening tool in the current study population. The major determining factor for which tool to recommend is the clinical setting where the tool is implemented, i.e. the availability of a computer-based alert system or not.

## Supplementary information

**Additional files 1-5.**

## Data Availability

The data that support the findings of this study are available from Karolinska Institutet Södersjukhuset but restrictions apply to the availability of these data, which were used under license for the current study, and so are not publicly available. Data are however available from the authors upon reasonable request and with permission of Karolinska Institutet Södersjukhuset.

## Abbreviations

AUC: Area Under the receiver operating Curve
GCS: Glasgow Coma Scale
CI: Confidence Interval
PPV: Positive Predictive Value
NPV: Negative Predictive Value
NCT: National Clinical Registration
ED: Emergency Department
suPAR: soluble urokinase Plasminogen Activator Receptor
HBP: Heparin Binding Protein
POC: Point-Of-Care
CRF: Case Report Form
FRAPP: framtida IT-plattform för prehospital vård i Stockholms läns landsting
ICD: International Classification of Diseases
SOFA: Sequential Organ Failure Assessment
PaO2: Partial pressure of Oxygen
FiO2: Fraction of inspired Oxygen
SPSS: Statistical Package for the Social Sciences
IQR: Interquartile Range
NEWS: National Early Warning Score
SIRS: Systemic Inflammatory Response Syndrome
ROC: Receiver Operating Characteristic
OR: Odds Ratio
CHAID: CHi-squared Automatic Interaction Detection
LR: Likelihood Ratio
STARD: Standards for the Reporting of Diagnostic accuracy studies
