# Supplementary material for: The predictive value of variables measurable in the ambulance and the development of the Predict Sepsis screening tools: a prospective cohort study
Source: Scand J Trauma Resusc Emerg Med. 2020 Jun 25;28:59. doi: 10.1186/s13049-020-00745-6 (PMC7318751; doi:10.1186/s13049-020-00745-6)
Supplement: Supplementary file 1 — Additional files 1-5. [file 13049_2020_745_MOESM1_ESM.docx]

**Additional files**

Supplement to: Wallgren U, Sjölin J, Järnbert-Pettersson H, Kurland L. **The predictive value of variables measurable in the ambulance and the development of the Predict Sepsis screening tools: a prospective cohort study**

**Table of Contents**

| Additional files | Content | Page | |
| --- | --- | --- | --- |
| 1 | Description of handling and analyses of point-of-care (POC) tests | | 2 |
| 2 | Definition of infection | | 3-4 |
| 3 | Prevalence of sepsis within categories | | 5-9 |
| 4 | Individual ROC curves for the 17 variables that showed a significant association with sepsis in the univariable analysis | | 10 |
| 5 | Models predictive of sepsis | | 11-13 |
| 6 | References for additional files | | 14 |

**Additional file 1. Description of handling and analyses of point-of-care (POC) tests.**

Blood was drawn in the ambulance for four POC-tests; P-glucose, P-lactate, P-HBP and P-suPAR.

*P-Glucose* was analyzed in the ambulance, in accordance with current ambulance guidelines^1^, using Contour® Blood Glucose Meter; Bayer, Basel, Switzerland.

The ED nurse receiving the ambulance collected the remaining blood tests. The samples were centrifuged and frozen at −70 °C until analysis. P-lactate and P-SuPAR were analyzed at Karolinska University Hospital study laboratory, Solna.

*P-Lactate* was analyzed in accordance with standard procedures at Karolinska University Hospital study laboratory, Solna.

*P-HBP*-samples were stored at Örebro Medicinska Biobank until analyses by the Inflammatory Response and Infection Susceptibility Centre (iRISC)^2^ laboratory in Örebro. Levels of P-HBP were determined using a commercial ELISA assay (Axis-Shield Diagnostics Ltd, Scotland) according to the manufacturer’s instructions. HBP was quantified based on a standard curve of known concentrations ranging from 0-200 ng/mL, where the lowest detection limit of the assay was 5.9 ng/mL. Samples were run in duplicates; the mean CV was 7.2%. Values below 5,9 or above 200 ng/mL were registered as 5,9 and 200 ng/mL respectively.

*P-suPAR* levels were determined in duplicate samples using a commercial enzyme-linked immunosorbent assay (ELISA) (suPARnostic® Standard kit; ViroGates A/S, Birkerød, Denmark) according to the manufacturer’s instructions. The linearity of this assay is comprised between 2.0 and 15.6 ng/mL, and the total imprecision, expressed as the coefficient of variation (CV %), ranges from 2.3 to 6.0 %. Values below 1.2 ng/mL or above 20.8 ng/mL were registered as 1.2 and 20.8 ng/mL respectively.

**Additional file 2. Definition of infection^α^**

| **Fever/ Chills** | **fever/chills as reported by patient or relatives / documentation that the patient feels feverish or a measured temperature >38.0°C.** |
| --- | --- |
| **Diarrhoea** | **in combination with other signs consistent with infection*.** |
| **Vomiting** | **vomiting in combination with other signs consistent with infection*** and without other obvious causes such as cerebrovascular lesion, new medication known to cause vomiting, benign postural vertigo, myocardial infarction or after head trauma. |
| **Pneumonia** | **current treatment for pneumonia *or* cough/chest pain /increased sputum/breathing difficulties in combination with other signs consistent with infection* *or* a low oxygen saturation without other cause.** If the patient suffered from isolated respiratory distress and had other, more likely causes, the patient was not considered to have a suspected pneumonia. Neither was Cheyne-Stokes respiration in combination with unconsciousness considered as pneumonia. |
| **Abdominal pain/ distension** | **abdominal pain/ tenderness during physical examination or a tense, distended or bloated abdomen in combination with other signs consistent with infection*.** |
| **Urinary tract infection** | **current treatment for urinary tract infection *or* cloudy urine/foul-smelling urine/new onset hematuria without trauma *or* dysuria/new difficulties to empty bladder *or* tenderness over the kidneys or urinary bladder on physical examination, in combination with other signs consistent with infection*.** |
| **Wound infection** | **bad smelling/purulent/black/deteriorated/exuding wounds.** Patients with chronic wounds without statement of deterioration were not considered to have a wound infection. |
| **Septic arthritis** | **red/ swollen/ warm/ painful joint without trauma or other, more likely, causes** (such as, for instance known metastasis/fracture) |
| **Meningitis** | **headache in combination with neck stiffness / photophobia/ a reduced level of consciousness and fever.** |
| **Cellulitis/ Soft tissue infection** | **red/ swollen/ warm/ painful soft tissue without trauma** including ongoing ab treatment for erysipelas |
| **Infected indwelling device** | **redness/purulent secretions or pain at site of venous catheters *or* signs consistent with infection* after catheterization within the last days.** |
| **Otitis** | **new-onset ear ache, without trauma, in combination with other signs consistent with infection*.** |
| **Tonsillitis/ Epiglottitis** | **new-onset throat pain, without trauma, in combination with other signs consistent with infection*.** |
| **Documentation of fever/ infection** | **Documentation of fever/ infection (infection, urinary tract infection, pneumonia, meningitis, flu, gastroenteritis.** Also, cholangitis with antibiotic treatment/ peritonitis with antibiotics were included**) / sepsis (sepsis, septic, urosepsis, septicaemia)** |
| **Drawn blood cultures** | **Blood cultures ordered by ED doctor** |
| **Antibiotics ordered** | **Antibiotics ordered by ED doctor** |
| **Increased C-reactive protein** | **CRP>100 mg/L^3^ without other cause, and in combination with other signs consistent with infection*** |
| ^α^ applied in addition to SOFA score to assess outcome sepsis.  *fever/chills, new-onset weakness/ malaise/ nausea/vomiting/ altered mental status/ hypotension.  °C=degree Celsius. | |

**Additional file 3. Prevalence of sepsis within categories.**

**Prevalence of sepsis within categories, 8-10 categories-variables.**

Colors indicate categories merged in the next step of categorization based on similarities with respect to sepsis prevalence within the categories, ORs and CIs for each category.

|  | | Outcome Sepsis in accordance with Sepsis-3 | | | |
| --- | --- | --- | --- | --- | --- |
|  |  | no | | yes | |
|  |  | Count | Row n% | Count | Row n% |
| RR ambulance,10 categories | <= 8 | 0 | 0,0% | 2 | 100,0% |
|  | 9 - 11 | 2 | 100,0% | 0 | 0,0% |
|  | 12 - 16 | 78 | 71,6% | 31 | 28,4% |
|  | 17 - 20 | 103 | 64,4% | 57 | 35,6% |
|  | 21 - 24 | 53 | 61,6% | 33 | 38,4% |
|  | 25 - 29 | 47 | 51,6% | 44 | 48,4% |
|  | 30 - 35 | 20 | 34,5% | 38 | 65,5% |
|  | 36 - 40 | 15 | 44,1% | 19 | 55,9% |
|  | 41 - 45 | 1 | 33,3% | 2 | 66,7% |
|  | 46+ | 0 | 0,0% | 3 | 100,0% |
| HR ambulance, 10 categories | <= 50 | 1 | 100,0% | 0 | 0,0% |
|  | 51 - 90 | 155 | 60,5% | 101 | 39,5% |
|  | 91 - 100 | 65 | 69,1% | 29 | 30,9% |
|  | 101 - 110 | 56 | 59,6% | 38 | 40,4% |
|  | 111 - 120 | 22 | 44,0% | 28 | 56,0% |
|  | 121 - 130 | 15 | 42,9% | 20 | 57,1% |
|  | 131 - 140 | 7 | 58,3% | 5 | 41,7% |
|  | 141 - 145 | 0 | 0,0% | 2 | 100,0% |
|  | 146 - 150 | 0 | 0,0% | 4 | 100,0% |
|  | 151+ | 0 | 0,0% | 2 | 100,0% |
| SpO2 ambulance,10 categories | <= 79 | 5 | 33,3% | 10 | 66,7% |
|  | 80 - 81 | 5 | 41,7% | 7 | 58,3% |
|  | 82 - 83 | 3 | 33,3% | 6 | 66,7% |
|  | 84 - 85 | 8 | 53,3% | 7 | 46,7% |
|  | 86 - 87 | 5 | 35,7% | 9 | 64,3% |
|  | 88 - 89 | 16 | 38,1% | 26 | 61,9% |
|  | 90 - 91 | 21 | 42,9% | 28 | 57,1% |
|  | 92 - 93 | 40 | 52,6% | 36 | 47,4% |
|  | 94 - 95 | 69 | 65,1% | 37 | 34,9% |
|  | 96+ | 149 | 70,6% | 62 | 29,4% |
| SBP ambulance,10 categories | <= 80 | 3 | 27,3% | 8 | 72,7% |
|  | 81 - 85 | 3 | 60,0% | 2 | 40,0% |
|  | 86 - 90 | 4 | 40,0% | 6 | 60,0% |
|  | 91 - 95 | 2 | 25,0% | 6 | 75,0% |
|  | 96 - 100 | 5 | 25,0% | 15 | 75,0% |
|  | 101 - 105 | 6 | 46,2% | 7 | 53,8% |
|  | 106 - 110 | 20 | 47,6% | 22 | 52,4% |
|  | 111 - 120 | 45 | 57,7% | 33 | 42,3% |
|  | 121 - 150 | 151 | 63,7% | 86 | 36,3% |
|  | 151+ | 81 | 65,3% | 43 | 34,7% |
| GCS ambulance, 8 categories | <= 6 | 0 | 0,0% | 1 | 100,0% |
|  | 7 - 8 | 0 | 0,0% | 2 | 100,0% |
|  | 9 - 10 | 0 | 0,0% | 1 | 100,0% |
|  | 11 - 11 | 1 | 16,7% | 5 | 83,3% |
|  | 12 - 12 | 1 | 16,7% | 5 | 83,3% |
|  | 13 - 13 | 0 | 0,0% | 10 | 100,0% |
|  | 14 - 14 | 36 | 40,4% | 53 | 59,6% |
|  | 15+ | 269 | 65,0% | 145 | 35,0% |
| Temp ambulance,10 categories | <= 35,0 | 1 | 100,0% | 0 | 0,0% |
|  | 35,1 - 35,4 | 2 | 66,7% | 1 | 33,3% |
|  | 35,5 - 35,9 | 6 | 100,0% | 0 | 0,0% |
|  | 36,0 - 38,0 | 161 | 72,2% | 62 | 27,8% |
|  | 38,1 - 38,5 | 53 | 58,2% | 38 | 41,8% |
|  | 38,6 - 39,0 | 40 | 46,5% | 46 | 53,5% |
|  | 39,1 - 39,5 | 34 | 45,3% | 41 | 54,7% |
|  | 39,6 - 40,0 | 18 | 43,9% | 23 | 56,1% |
|  | 40,1 - 40,5 | 5 | 31,3% | 11 | 68,8% |
|  | 40,6+ | 1 | 20,0% | 4 | 80,0% |
| P-Glucose ambulance,10 categories | <= 6,5 | 69 | 66,3% | 35 | 33,7% |
|  | 6,6 - 7,7 | 88 | 61,1% | 56 | 38,9% |
|  | 7,8 - 8,9 | 50 | 54,3% | 42 | 45,7% |
|  | 9,0 - 10,1 | 32 | 55,2% | 26 | 44,8% |
|  | 10,2 - 11,3 | 18 | 60,0% | 12 | 40,0% |
|  | 11,4 - 12,5 | 15 | 57,7% | 11 | 42,3% |
|  | 12,6 - 13,7 | 8 | 42,1% | 11 | 57,9% |
|  | 13,8 - 14,9 | 12 | 63,2% | 7 | 36,8% |
|  | 15,0 - 16,1 | 3 | 75,0% | 1 | 25,0% |
|  | 16,2+ | 9 | 50,0% | 9 | 50,0% |
| P-Lactate ambulance,10 categories | <= 1,0 | 39 | 68,4% | 18 | 31,6% |
|  | 1,1 - 2,0 | 177 | 63,0% | 104 | 37,0% |
|  | 2,1 - 3,0 | 63 | 55,3% | 51 | 44,7% |
|  | 3,1 - 4,0 | 23 | 50,0% | 23 | 50,0% |
|  | 4,1 - 5,0 | 4 | 26,7% | 11 | 73,3% |
|  | 5,1 - 6,0 | 4 | 44,4% | 5 | 55,6% |
|  | 6,1 - 7,0 | 1 | 20,0% | 4 | 80,0% |
|  | 7,1 - 8,0 | 0 | 0,0% | 3 | 100,0% |
|  | 8,1 - 9,0 | 0 | 0,0% | 2 | 100,0% |
|  | 9,1+ | 0 | 0,0% | 4 | 100,0% |
| P-SuPAR ambulance,10 categories | <= 1,99 | 8 | 80,0% | 2 | 20,0% |
|  | 2,00 - 3,99 | 120 | 69,0% | 54 | 31,0% |
|  | 4,00 - 5,99 | 99 | 57,9% | 72 | 42,1% |
|  | 6,00 - 7,99 | 54 | 60,0% | 36 | 40,0% |
|  | 8,00 - 9,99 | 19 | 39,6% | 29 | 60,4% |
|  | 10,00 - 11,99 | 9 | 42,9% | 12 | 57,1% |
|  | 12,00 - 13,99 | 4 | 25,0% | 12 | 75,0% |
|  | 14,00 - 15,99 | 1 | 25,0% | 3 | 75,0% |
|  | 16,00 - 17,99 | 0 | 0,0% | 3 | 100,0% |
|  | 18,00+ | 0 | 0,0% | 1 | 100,0% |
| HBP_ambulance_10_categories | <= 5,90 | 109 | 73,2% | 40 | 26,8% |
|  | 5,91 - 14,99 | 85 | 60,3% | 56 | 39,7% |
|  | 15,00 - 22,99 | 32 | 46,4% | 37 | 53,6% |
|  | 23,00 - 29,99 | 25 | 56,8% | 19 | 43,2% |
|  | 30,00 - 63,99 | 33 | 46,5% | 38 | 53,5% |
|  | 64,00 - 97,99 | 14 | 46,7% | 16 | 53,3% |
|  | 98,00 - 131,99 | 1 | 20,0% | 4 | 80,0% |
|  | 132,00 - 165,99 | 1 | 33,3% | 2 | 66,7% |
|  | 166,00 - 199,99 | 1 | 50,0% | 1 | 50,0% |
|  | 200,00+ | 4 | 36,4% | 7 | 63,6% |
|  | Total | 305 | 58,1% | 220 | 41,9% |

**Prevalence of sepsis within categories, 3-4 categories-variables.**

Colors indicate categories merged in the next step of categorization based on similarities with respect to sepsis prevalence within the categories, ORs and CIs for each category.

|  | | Outcome Sepsis in accordance with Sepsis-3 | | | |
| --- | --- | --- | --- | --- | --- |
|  |  | no | | yes | |
|  |  | Count | Row n% | Count | Row n% |
| RR ambulance, 3 categories | <= 24 | 236 | 65,7% | 123 | 34,3% |
|  | 25 - 29 | 47 | 51,6% | 44 | 48,4% |
|  | 30+ | 36 | 36,7% | 62 | 63,3% |
| SpO2 ambulance, 3 categories | <= 83 | 13 | 36,1% | 23 | 63,9% |
|  | 84 - 93 | 90 | 45,9% | 106 | 54,1% |
|  | 94+ | 218 | 68,8% | 99 | 31,2% |
| HR ambulance, 3 categories | <= 90 | 156 | 60,7% | 101 | 39,3% |
|  | 91 - 110 | 121 | 64,4% | 67 | 35,6% |
|  | 111+ | 44 | 41,9% | 61 | 58,1% |
| SBP ambulance, 3 categories | <= 100 | 17 | 31,5% | 37 | 68,5% |
|  | 101 - 110 | 26 | 47,3% | 29 | 52,7% |
|  | 111+ | 277 | 63,1% | 162 | 36,9% |
| GCS ambulance, 3 categories | <= 13 | 2 | 7,7% | 24 | 92,3% |
|  | 14 - 14 | 36 | 40,4% | 53 | 59,6% |
|  | 15+ | 269 | 65,0% | 145 | 35,0% |
| Temp ambulance, 4 categories | <= 38,0 | 170 | 73,0% | 63 | 27,0% |
|  | 38,1 - 38,5 | 53 | 58,2% | 38 | 41,8% |
|  | 38,6 - 40,0 | 92 | 45,5% | 110 | 54,5% |
|  | 40,1+ | 6 | 28,6% | 15 | 71,4% |
| P-Glucose ambulance, 3 categories | <= 6,5 | 69 | 66,3% | 35 | 33,7% |
|  | 6,6 - 12,5 | 203 | 58,0% | 147 | 42,0% |
|  | 12,6+ | 32 | 53,3% | 28 | 46,7% |
| P-Lactate ambulance, 4 categories | <= 2,0 | 216 | 63,9% | 122 | 36,1% |
|  | 2,1 - 4,0 | 86 | 53,8% | 74 | 46,3% |
|  | 4,1 - 6,0 | 8 | 33,3% | 16 | 66,7% |
|  | 6,1+ | 1 | 7,1% | 13 | 92,9% |
| P-SuPAR ambulance, 4 categories | <= 3,99 | 128 | 69,6% | 56 | 30,4% |
|  | 4,00 - 7,99 | 153 | 58,6% | 108 | 41,4% |
|  | 8,00 - 11,99 | 28 | 40,6% | 41 | 59,4% |
|  | 12,00+ | 5 | 20,8% | 19 | 79,2% |
| HBP_ambulance_3_categories | <= 14,99 | 194 | 66,9% | 96 | 33,1% |
|  | 15,00 - 97,99 | 104 | 48,6% | 110 | 51,4% |
|  | 98,00+ | 7 | 33,3% | 14 | 66,7% |
|  | Total | 305 | 58,1% | 220 | 41,9% |

**Prevalence of sepsis within categories, final 2-3 categories-variables.**

|  | | Outcome Sepsis in accordance with Sepsis-3 | | | |
| --- | --- | --- | --- | --- | --- |
|  |  | no | | yes | |
|  |  | Count | Row n% | Count | Row n% |
| FINAL_RR ambulance, 2 categories | <= 24 | 236 | 65,7% | 123 | 34,3% |
|  | 25+ | 83 | 43,9% | 106 | 56,1% |
| FINAL_SpO2 ambulance, 2 categories | <= 93 | 103 | 44,4% | 129 | 55,6% |
|  | 94+ | 218 | 68,8% | 99 | 31,2% |
| FINAL_HR ambulance, 2 categories | <= 110 | 277 | 62,2% | 168 | 37,8% |
|  | 111+ | 44 | 41,9% | 61 | 58,1% |
| FINAL_SBP ambulance, 2 categories | <= 100 | 17 | 31,5% | 37 | 68,5% |
|  | 101+ | 303 | 61,3% | 191 | 38,7% |
| FINAL_GCS ambulance, 2 categories | <= 14 | 38 | 33,0% | 77 | 67,0% |
|  | 15+ | 269 | 65,0% | 145 | 35,0% |
| FINAL_Temp ambulance, 3 categories | <= 38,0 | 170 | 73,0% | 63 | 27,0% |
|  | 38,1 - 38,5 | 53 | 58,2% | 38 | 41,8% |
|  | 38,6+ | 98 | 43,9% | 125 | 56,1% |
| FINAL_P-Glucose ambulance, 2 categories | <= 6,5 | 69 | 66,3% | 35 | 33,7% |
|  | 6,6+ | 235 | 57,3% | 175 | 42,7% |
| FINAL_P-Lactate ambulance, 3 categories | <= 2,0 | 216 | 63,9% | 122 | 36,1% |
|  | 2,1 - 4,0 | 86 | 53,8% | 74 | 46,3% |
|  | 4,1+ | 9 | 23,7% | 29 | 76,3% |
| FINAL_P-SuPAR ambulance, 3 categories | <= 3,99 | 128 | 69,6% | 56 | 30,4% |
|  | 4,00 - 7,99 | 153 | 58,6% | 108 | 41,4% |
|  | 8,00+ | 33 | 35,5% | 60 | 64,5% |
| FINAL_HBP ambulance, 2 categories | <= 14,99 | 194 | 66,9% | 96 | 33,1% |
|  | 15,00+ | 111 | 47,2% | 124 | 52,8% |

RR=Respiratory rate, SpO2= Peripheral Oxygen saturation, HR=Heart Rate, SBP=Systolic Blood Pressure, GCS=Glasgow Coma Scale, Temp=Temperature, SuPAR=Soluble urokinase Plasminogen Activating Receptor, HBP=Heparin Binding Protein.

**Additional file 4. Individual ROC curves for the 17 variables that showed a significant association with sepsis in the univariable analysis.**


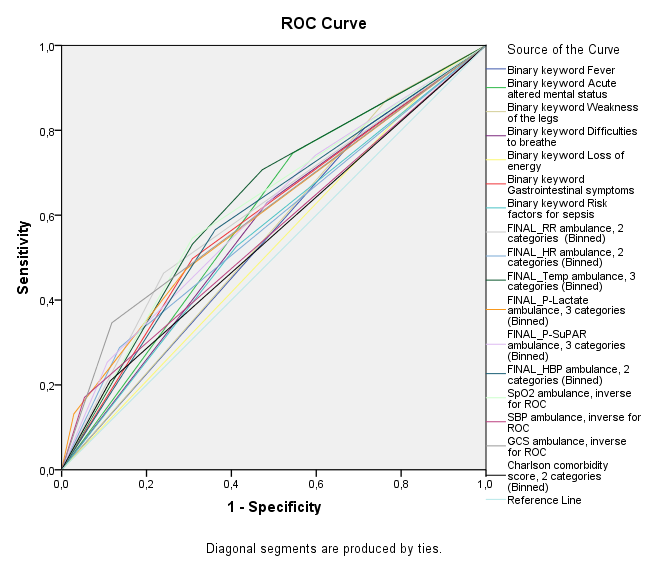


ROC=Receiver Operating Curves, RR=Respiratory rate, HR=Heart Rate, Temp=Temperature, SuPAR=Soluble urokinase Plasminogen Activating Receptor, HBP=Heparin Binding Protein, SpO2= Peripheral Oxygen saturation, SBP=Systolic Blood Pressure, GCS=Glasgow Coma Scale.

**Additional file 5. Models predictive of sepsis.**

| **Model** | **Number of included variables** | **Variables included and scores in final models** | **Total score considered positive for suspected sepsis** | **Method used to develop the model** | **Hosmer and Lemeshow for goodness of fit of the model,**  **P-value** | **AUC for the model before introducing scores for included variables**  **(95%CI)*** |
| --- | --- | --- | --- | --- | --- | --- |
| Model 1  14 variables^α^ including HBP and suPAR | 14 | *Acute altered mental status*  *Weakness of the legs*  *Breathing difficulties*  *Gastrointestinal symptoms*  *Risk factors for sepsis*  RR>24  HR>110  Temp 38.1-38.5 or >38.5  SpO2<94  SBP≤100  GCS<15  P-Lactate 2.1-4.0 or >4.0  P-suPAR 4.0-7.99 or ≥8  P-HBP≥15 | - | Variables significant in univariable analysis and with a significant P-value for AUC and AUC>0.600, including HBP and suPAR. | 0.314 | 0.81  (0.77-0.84) |
| Model 2  12 variables^α^ excluding  HBP and suPAR | 12 | *Acute altered mental status*  *Weakness of the legs*  *Breathing difficulties*  *Gastrointestinal symptoms*  *Risk factors for sepsis*  RR>24  HR>110  Temp 38.1-38.5 or >38.5  SpO2<94  SBP≤100  GCS<15  P-Lactate 2.1-4.0 or >4.0 | - | Variables significant in univariable analysis and with significant P-value for AUC and AUC>0.600, excluding HBP and suPAR. | 0.550 | 0.80  (0.76-0.84) |
| Model 3  =**Predict Sepsis screening tool 1** | 6 | *Acute altered mental status*=1  *Gastrointestinal symptoms*=1  SBP≤100=2  GCS<15=2  Temp 38.1-38.5=1  Temp >38.5= 2  P-Lactate>4.0=2 | ≥2 | Variables with significant association with sepsis in multivariable logistic regression. | 0.850 | 0.79  (0.75-0.83) |
| Model 4  As Model 3 but without P-Lactate | 5 | *Acute altered mental status*=1  *Gastrointestinal symptoms*=1  SBP≤100=2  GCS<15=2  Temp 38.1-38.5=1  Temp >38.5= 2 | ≥2 | Variables with significant association with sepsis in multivariable logistic regression without P-Lactate. | 0.936 | 0.78  (0.74-0.82) |
| Model 5  As Model 3 but with a combined variable for keyword Acute altered mental status and GCS<15 with score=2 | 5 | *Acute altered mental status and/or GCS<15*=2  *Gastrointestinal symptoms*=1  SBP≤100=2  Temp 38.1-38.5=1  Temp>38.5= 2  P-Lactate>4.0=2 | ≥2 | Variables with significant association with sepsis in multivariable logistic regression but with a combined variable for keyword Acute altered mental status and GCS<15. | 0.311 | 0.76  (0.71-0.80) |
| Model 6  As model 5 but without P-Lactate | 4 | *Acute altered mental status and/or GCS<15*=2  *Gastrointestinal symptoms*=1  SBP≤100=2  Temp 38.1-38.5=1  Temp>38.5= 2 | ≥2 | As model 5 but without P-Lactate. | 0.919 | 0.74  (0.70-0.79) |
| Model 7  As model 5 but with score=1 for the combined variable for keyword Acute altered mental status and GCS<15 | 5 | *Acute altered mental status and/or GCS<15*=1  *Gastrointestinal symptoms*=1  SBP≤100=2  Temp 38.1-38.5=1  Temp>38.5= 2  P-Lactate>4.0=2 | ≥2 | Variables with significant association with sepsis in multivariable logistic regression but with a combined variable for keyword Acute altered mental status and GCS<15. | 0.311 | 0.76  (0.71-0.80) |
| **Predict Sepsis screening tool 2**  As model 7 but without P-Lactate | 4 | *Acute altered mental status and/or GCS<15*=1  *Gastrointestinal symptoms*=1  SBP≤100=2  Temp 38.1-38.5=1  Temp>38.5= 2 | ≥2 | As model 7 but without P-Lactate. | 0.919 | 0.74  (0.70-0.79) |
| **Predict sepsis screening tool** **3** utilizing the calculated categories for vital signs | 6 | RR>24=1  SpO2<94=1  HR>110=1  SBP≤100=2  GCS <15=2  Temp 38.1-38.5=1  Temp>38.5= 2 | ≥2 | Vital signs significant in univariable logistic regression. | 0.231 | 0.77  (0.73-0.81) |
| AUC=Area Under the (receiver operating) Curve, CI=Confidence Interval, HBP=Heparin Binding Protein, suPAR=Soluble urokinase Plasminogen Activating Receptor, RR=Respiratory rate, HR=Heart rate, Temp=Temperature, SpO2=Peripheral Oxygen saturation, SBP=Systolic Blood Pressure, GCS=Glasgow Coma Scale.  *AUC values for the final models after introducing scores are presented in Table 3 in the manuscript.  **^α^**Scores for individual included variables and total scores were not developed for these models.  *Curved text indicates keywords related to medical history.*  **Bolded text indicates the models chosen as the Predict Sepsis screening tools.** | | | | | | |

**6. References for additional files.**

1. Medical guidelines for the ambulance care. In: Stockholm City Council; 2015.

2. Inflammatory Response and Infection Susceptibility Centre (iRiSC) Örebro University; 2018. <https://www.oru.se/english/research/research-environments/mh/inflammatory-response-and-infection-susceptibility-centre-irisc/>. Accessed May 2, 2019.

3. Vårdprogram Sepsis och septisk chock – tidig identifiering och initial handläggning 2018. Swedish Society of Infectious Medicine; 2018. <http://infektion.net/wp-content/uploads/2018/06/revision-sepsis-och-septisk-chock-180626.pdf>. Accessed February 15, 2019.
